# A Smell That Causes Seizure

**DOI:** 10.1371/journal.pone.0041899

**Published:** 2012-07-27

**Authors:** Minh Q. Nguyen, Nicholas J. P. Ryba

**Affiliations:** Taste and Smell Section, Laboratory of Sensory Biology, National Institute of Dental and Craniofacial Research, National Institutes of Health, Bethesda, Maryland, United States of America; Duke University, United States of America

## Abstract

In mammals, odorants are detected by a large family of receptors that are each expressed in just a small subset of olfactory sensory neurons (OSNs). Here we describe a strain of transgenic mice engineered to express an octanal receptor in almost all OSNs. Remarkably, octanal triggered a striking and involuntary phenotype in these animals, with passive exposure regularly inducing seizures. Octanal exposure invariably resulted in widespread activation of OSNs but interestingly seizures only occurred in 30–40% of trials. We hypothesized that this reflects the need for the olfactory system to filter strong but slowly-changing backgrounds from salient signals. Therefore we used an olfactometer to control octanal delivery and demonstrated suppression of responses whenever this odorant is delivered slowly. By contrast, rapid exposure of the mice to octanal induced seizure in every trial. Our results expose new details of olfactory processing and provide a robust and non-invasive platform for studying epilepsy.

## Introduction

Our senses evolved to provide useful information about the environment. Consequently, stimuli received outside their normal context often elicit undesirable effects: e.g. a bright flash of light temporarily blinds an individual adapted to dim conditions. Similarly, a sudden loud noise in a quiet setting can seem painful even though the same sound in a noisy environment is barely noticeable. Olfaction detects and distinguishes odorants through changes in activity of olfactory sensory neurons (OSNs) and provides mammals with exquisite ability to separate novel chemical signatures from a more constant but sometimes intense background. What would simultaneous activation of all OSNs smell like? And what would the consequences be?

Mammalian olfaction is initiated by activation of olfactory sensory neurons (OSNs) in the main olfactory epithelium (MOE) through detection of odorants by a large family of odorant receptors (ORs) [1], [2]. Each OSN expresses a single OR [3], [4] and neurons expressing the same OR project to stereotypic sites in the main olfactory bulb (MOB) forming structures known as glomeruli [5]–[8]. The MOB output neurons, mitral and tufted (M/T) cells, send apical dendrites into a single glomerulus meaning M/T cells are tuned to respond to activation of a single OR [9]. The M/T cells innervate multiple regions of the brain with the piriform cortex being a major target [10]–[12]. Axons of the M/T cells associated with a single glomerulus project to a distributed array of pyramidal cells in the piriform cortex. Thus a particular odorant activates a sparse, idiosyncratic subpopulation of principal neurons scattered across the piriform cortex [13]–[15]. It has been suggested that an extensive recurrent excitatory network within the piriform cortex amplifies responses and results in the widespread representation of even minor olfactory stimulation [16], [17].

Recently, a mouse with a “monoclonal nose” expressing an acetophenone receptor (M71) in 95% of OSNs was reported [18]. Remarkably, activation of M71 could readily be detected at the level of the MOE in these mice but responses within the MOB were dramatically attenuated and the animals failed to detect acetophenone in behavioral assays. This suggests that inhibitory circuits within the olfactory system filter out common events occurring broadly within the MOB and thus increase the salience of changes in glomerular activity against even strong backgrounds of widespread OSN firing [18]. Here we have generated a different line of mice expressing a single OR in almost all OSNs and show that odorant exposure can evoke widespread brain activity and trigger seizures in these “Odor-induced Seizure”(OiS)-mice. We also demonstrate that concentration and rate of odorant delivery strongly influence the outcome in a predictable manner making OiS-mice a robust model for studying seizure.

## Results

**Figure 1:**
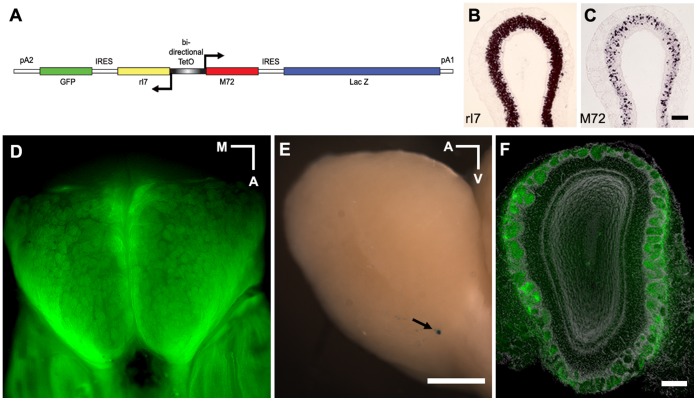
Expression of *rI7* and *M72* in OiS-mice. (A) Schematic representation of the bidirectional *TetO* construct used to express two different ORs (*rI7* and *M72*) from a single locus. *In situ* hybridization demonstrated that approx. 90% of OSNs expressed *rI7* (B) and only approx. 5% of OSNs expressed *M72* (C); more detail is presented in supplemental data (Fig. S1). The projection pattern of OSNs expressing *tgOR*s in OiS-animals was monitored using the co-expressed marker gene (D–F). The MOB exhibited a normal distribution and size range of glomeruli. Whole-mount fluorescence of the dorsal OB revealed rI7 and GFP-expressing neurons innervate nearly all glomeruli (D). Although the M72 transgene is expressed in ∼5% of mature OSNs in OiS-mice, staining for LacZ revealed very few glomerular targets for M72-expressing neurons (E, arrowed). The image shown (lateral view) is typical of LacZ staining in this line with most stained fibers restricted to the ventro-caudal region of the bulb. (This is quite different from LacZ staining in other *tgOR-LacZ*-lines where targeted glomeruli are generally very clearly labeled even when an equal number of MOE neurons express LacZ thus this does not reflect restriction of LacZ to the cell bodies of OSNs.) (F) GFP-fluorescence (green) in a coronal section through the MOB of an OiS-mouse counterstained with DAPI (gray) demonstrates that all glomeruli contained rI7 and GFP-positive fibers. Scale bars: B & C, 100 µm; D & E, 1 mm; F, 200 µm. Medial (M), Anterior (A), and Ventral (V) directions in whole-mount images are indicated.

We and others showed previously that by using the Tetracycline-controlled Tet-on/Tet-off system, endogenous OR gene regulation mechanisms can be bypassed to express any *tgOR* in a large number of OSNs [18], [19]. Here, we have engineered transgenic animals where two different *tgORs* (*rI7* and *M72* tagged with either an IRES-GFP or IRES-lacZ) were placed under the control of a single bidirectional *Tet-operator* (Fig. 1A). Mice carrying *tg(bi)OR* transgenes were crossed into a background expressing the tetracycline dependent transactivator (TTA) in all mature olfactory neurons (*OMP-TTA* knockin) [20] generating *OMP-tg(bi)OR* mice (see Methods for details). In one *OMP-tg(bi)OR* line, Line 319A (referred to as the OiS-line), an octanal receptor (*rI7*) was expressed in more than 90% of OSNs and an acetophenone receptor (*M72*) in a further ∼5% (Figs. 1, S1). Thus, the expression of *rI7* in OiS-mice resembles that of tgORs in the *OMP-tgM71* and *OMP-tgMOR28B* lines that were reported earlier [18], [21]. The anatomy of the MOB in OiS-mice is similar to controls and other OMP-tgOR lines [18], [21] with most glomeruli filled with *rI7-IRES-GFP* expressing primary afferents (GFP-fluorescence, Fig. 1D, F). Interestingly, although *M72-IRES-LacZ* was expressed in about 5% of OSNs, we observed very few lacZ positive fibers in the MOB (Fig. 1C, E).

Previously it has been reported that mice with global expression of the OR M71 cannot detect acetophenone, the M71 cognate ligand, although they still are able to detect other odorants [18]. Therefore we anticipated that the OiS-line would behave in a similar manner and began by testing mice in a standard habituation-dishabituation assay [22]. Our results (Fig. S2) suggest that this assay lacks sensitivity as control animals showed minimal responses to a range of odorants including octanal and acetophenone. Intriguingly, both control and OiS-animals spent no more time investigating octanal-scented filter paper than a filter paper spotted with mineral oil. Surprisingly, however, OiS mice regularly exhibited a dramatic and involuntary phenotype: out of 21 OiS-mice exposed to 1% octanal, three exhibited major tonic-clonic seizures, two exhibited moderate and two milder seizure-like effects (see below and Movies S1, S2 & S3). As expected, control mice (including mice carrying the *OiS-bicistronic tgOR* but no *TTA* driver) never exhibited seizure-like symptoms in the presence of octanal and OiS-animals never showed even mild effects that resembled seizure when exposed to a wide variety of other odorants including acetophenone (Movies S4 & S5). Finally, in trials where OiS-animals did not experience seizure, mice regularly investigated and even sniffed the filter paper (Movie S6).

Seizures followed a stereotyped progression that did not require mice to approach the odorant-scented filter paper. Strong seizures began within the first 15–20 s of a transgenic mouse being exposed to octanal, while weaker events were usually observed within the first minute of exposure. Symptoms were characterized by three stages: in the first, mice began to rapidly blink their eyes before retreating while losing control of their forelimbs; this phase typically lasted 10–15 s. The second stage began with mice rearing up onto their hind-legs while retracting their forepaws and evolved to mice foaming at the mouth over a period of 5–10 s. In the third phase, mice collapsed backwards before repeatedly rearing and collapsing again for a period of 15–30 s. After this, mice generally lay immotile for a period ranging from 10 s to 30 minutes before fully recovering. Animals exhibiting all these symptoms were classified as showing severe effects, whereas mild and moderate symptoms were ascribed to mice exhibiting only the first or first two stages of seizure, respectively.

We reasoned that if octanal was inducing seizure through the olfactory system, increasing the concentration of the odorant should increase the number and severity of events observed. Indeed, when 10% octanal was used as odorant both the fraction of mice displaying seizure and the severity of the symptoms increased (Table 1). We noted that individual mice exposed to this test multiple times did not show a particular pattern in their response: within the limits of our data all OiS-transgenic mice were equally likely to exhibit symptoms (and variable severity of seizure) each time they were exposed to octanal thus it is unlikely that genetic variability or level of transgene expression account for differences in response.

**Table 1:** Number of OiS-mice that experienced seizures when exposed to 1% or 10% octanal (see Movies S1, S2, S3, S4, S5, and S6 for examples of the different levels of seizure recorded here).

% octanal	delivery method	Number of OiS animals with seizure			
		strong	moderate	weak	Total seizure/total tested
1	passive	3 (14%)	2 (10%)	2 (10%)	7/21 (33%)
10	passive	8 (19%)	6 (14%)	3 (7%)	17/42 (40%)

**Figure 2:**
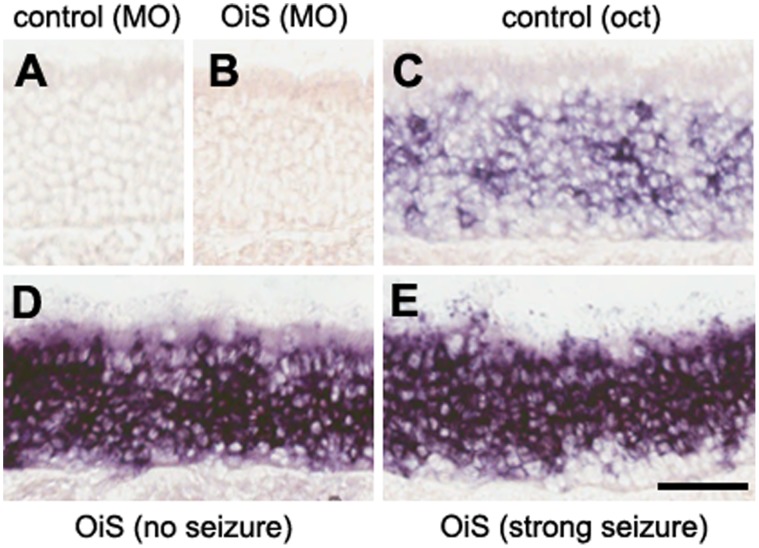
Octanal induced activation of OSNs in the MOE. *In situ* hybridization was used to monitor expression of *c-fos* as a measure of neuronal activity in the MOE. Control (A) and OiS- (B) mice showed essentially no *c-fos* expression after exposure to mineral oil. Exposure of mice to filter paper carrying a 20 µl drop of 10% octanal in mineral oil (MO) induced moderate *c-fos* expression in a subset (25–40%) of OSNs of control animals (C) and strong expression in the vast majority (≥90%) of OiS-line OSNs (D–E). No systematic differences in the intensity or density of *c-fos* staining were observed between individual exhibiting no response (D) or strong (E) seizures. Scale bar, 50 µm.

Another simple explanation for why OiS-mice exhibit variable responses to octanal could be that different proportions of OSNs are activated by odorant. However, we found no evidence supporting such a scenario when we examined *c-fos* expression as a measure of neuronal activity [23], [24]. Octanal exposure induced (predominantly weak) *c-fos* expression in a relatively large subset of OSNs of control animals (compare Fig. 2A, C), consistent with octanal activating multiple different endogenous ORs in the mouse [25]. As expected, OiS-mice exhibited much more pronounced OSN activity after octanal exposure. However, there was no detectable difference in the number of responding OSNs or the strength of *c-fos* expression between mice that experienced a seizure and those that showed no adverse response to octanal (Fig. 2D, E). Indeed, in all cases, essentially the entire population of OSNs now expressed high levels of *c-fos.* These results suggest that the variation in symptoms that we observed is more dependent on differences in signal processing in the MOB and higher brain centers than the overall extent of MOE-activation.

**Figure 3:**
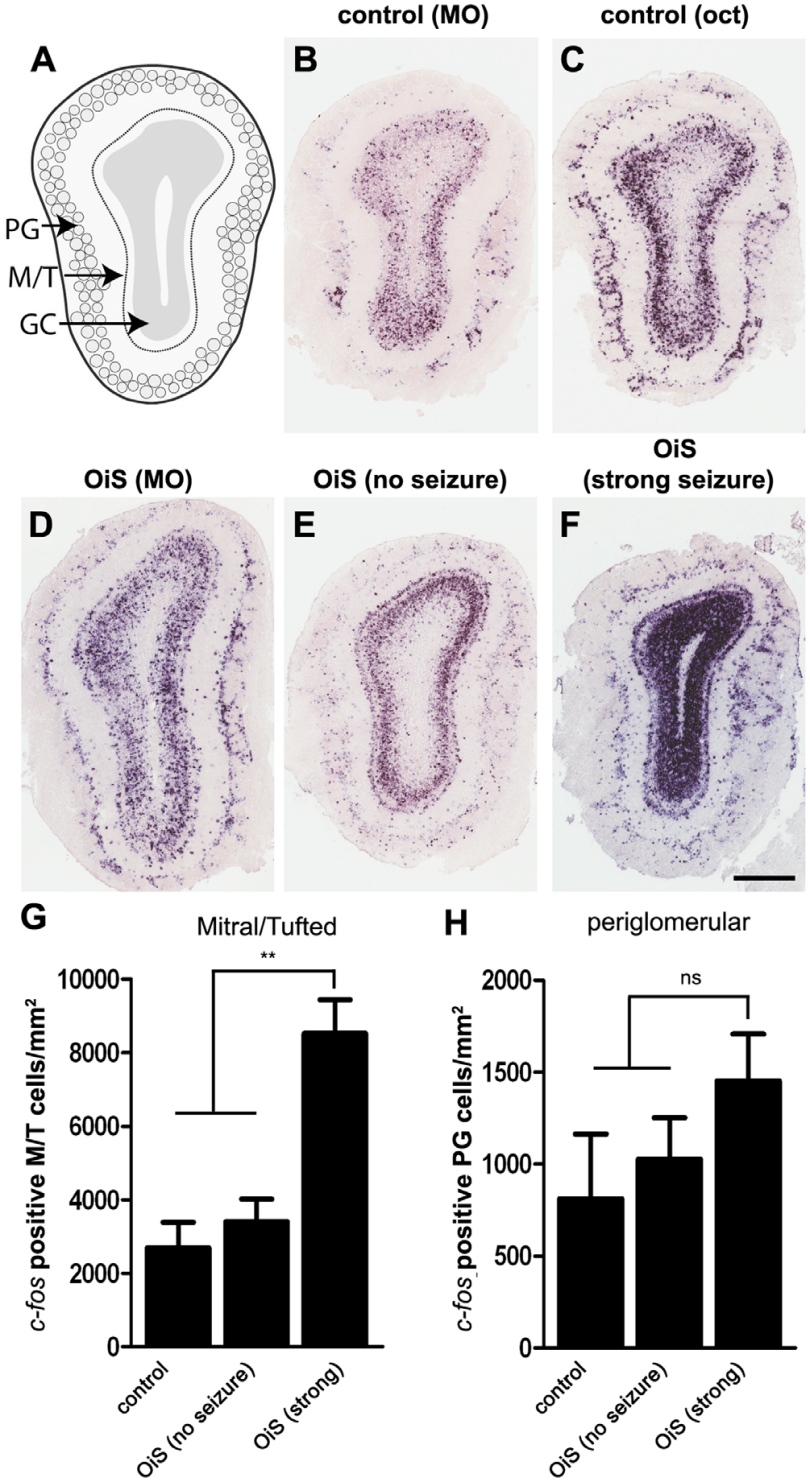
Neuronal activation in the MOB after octanal stimulation. (A) Schematic diagram of the main olfactory bulb (MOB) showing the location of periglomerular (PG), mitral and tufted (M/T), and granular (GC) cells. (B–F) Representative images from *in situ* hybridization of sections through the MOB using a *c-fos* probe: mineral oil exposure of control (B) and OiS- (D) mice revealed background *c-fos* expression that was concentrated in the granule cell layer. Only slight increases in *c-fos* staining were observed when control mice were (C) challenged with octanal. In contrast, octanal exposure resulted in dramatic differences in MOB neuronal activity of OiS-mice that reflected whether the mouse exhibited symptoms of seizure (E, F). Animals that did not exhibit any symptoms displayed a *c-fos* expression pattern (E) that resembled *c-fos* expression in control mice (B, C) or in OiS animals exposed to mineral oil (D). In contrast, OiS-mice that showed strong seizures exhibited a prominent increase in *c-fos* expression in the M/T but no significant change in activity of PG cells (see G, H for quantitation). In addition, octanal induced seizures were characterized by prominent labeling of the entire granule cell layer. Scale bars: B–F, 500 µm; data are mean ± s.e.m, n = 3 mice; **denotes *p*<0.01.

We next examined neuronal activity in the MOB again using *c-fos* induction to allow assessment of changes in activity throughout the bulb. Although immediate early gene expression provides a powerful approach to study large scale and global changes in neuron firing, its temporal resolution and sensitivity coupled with appreciable background mean that subtle changes in activity may be missed. Control animals exhibited appreciable neuronal activity in both periglomerular (PG) and granule cells both after exposure to carrier (mineral oil) and odorant (octanal, Fig. 3B, C). A similar pattern of *c-fos* expression was observed in OiS-mice exposed just to mineral oil (Fig. 3D). Notably, however, OiS-mice showed differences in *c-fos* induction that paralleled their seizure symptoms when challenged with octanal. Mice that showed no response did not experience a seizure were characterized by little or no increase in bulbar *c-fos* expression after octanal exposure (Fig. 3E, G, H). In contrast, mice exhibiting strong seizures displayed increased expression of *c-fos* in M/T cells (Fig. 3F, G) as well as very prominent activity in the vast majority of granule cells (Fig. 3F) but not in the inhibitory PG cells (Fig. 3F, H).

**Figure 4:**
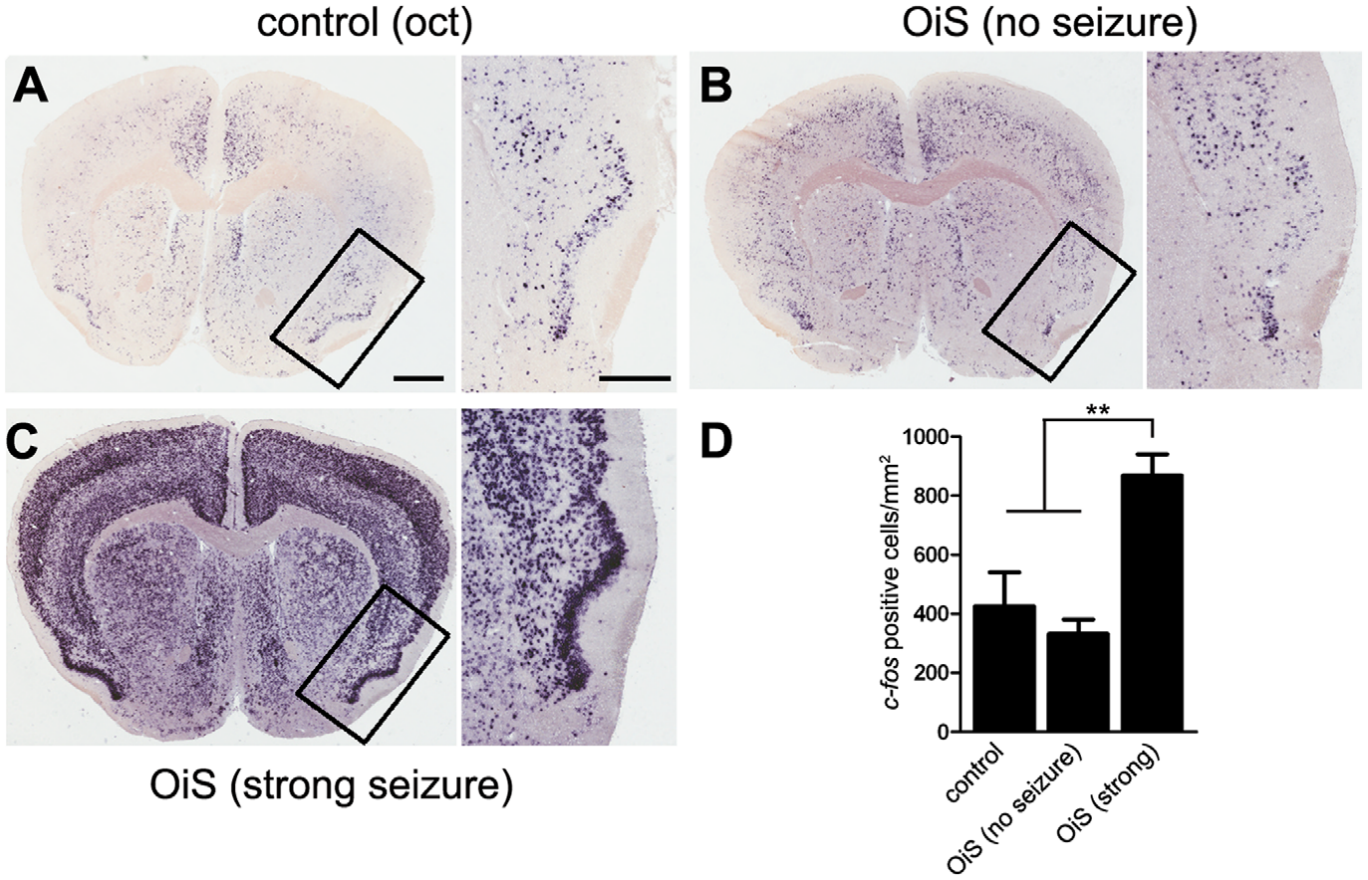
The spread of neuronal activity corresponds to severity of seizures in OiS-mice. *In situ* hybridization for *c-fos* was used to monitor the spread of neuronal activity in the piriform cortex and other regions of the brain (see also Fig. 5). Representative coronal sections at approx. Bregma +1.0 of control (A) and OiS-mouse brains (B, C) are shown; boxed area (piriform cortex) in each panel is shown magnified to the right. After exposure to 10% octanal, control mice showed *c-fos* expression in a sparse and randomly distributed population of cells in the piriform cortex and other regions of the brain (A). A similar pattern of neural activity was observed in OiS-mice if they exhibited no seizure-like symptoms (B). Mice exhibiting strong symptoms of seizures in response to octanal displayed a robust increase in *c-fos* expression in the piriform cortex with labeling of many neurons in all layers (C). Seizures also triggered massive neural activity in most other regions of the forebrain. (D) Quantitation of *c-fos* positive cells in the piriform cortex. Scale bars: A–C, 1 mm; 500 µm for magnified boxed area, data are mean ± s.e.m, n = 3 mice; **denotes *p*<0.01.

**Figure 5:**
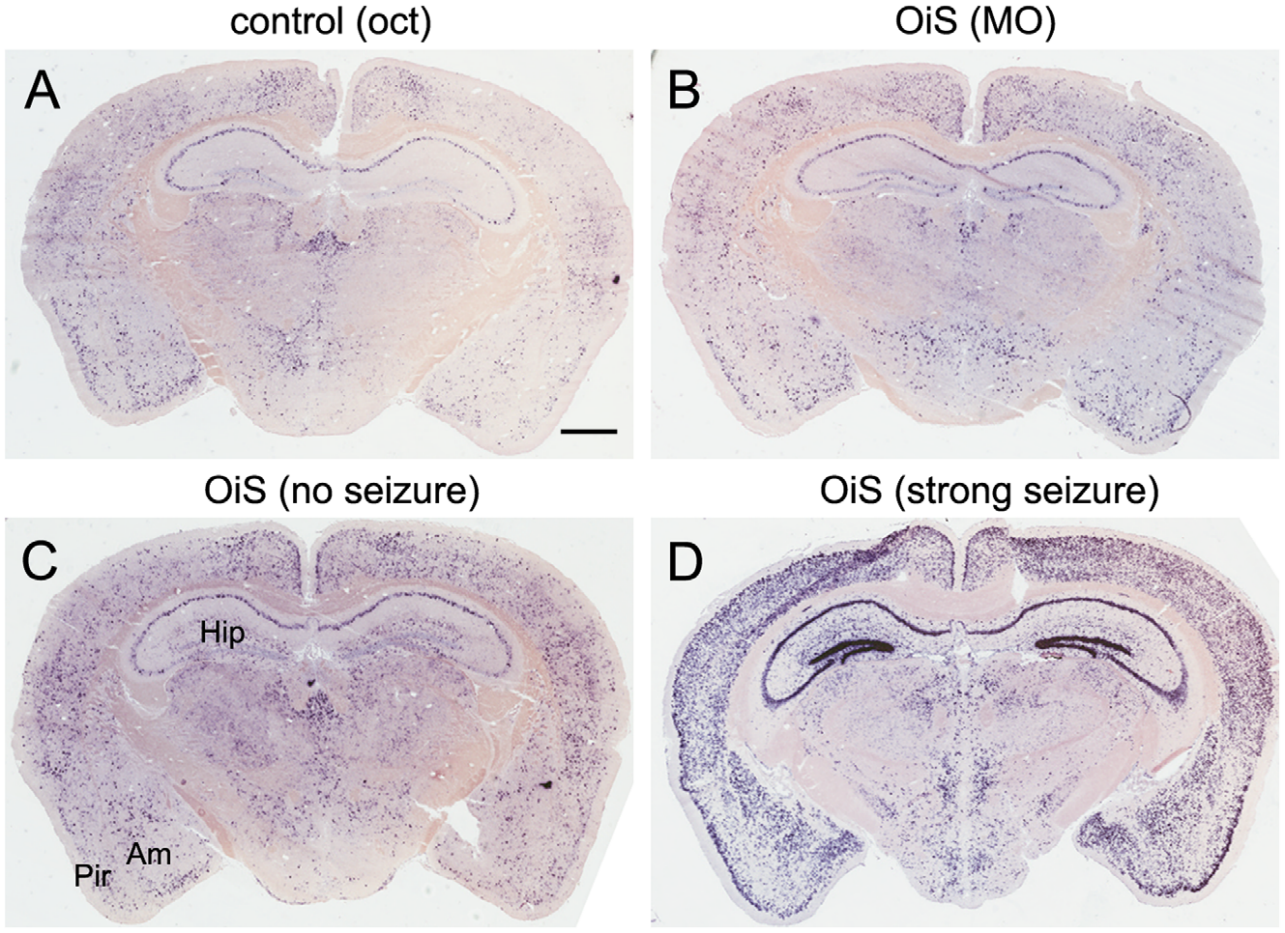
Odorant induced seizures in OiS-mice are tied to pronounced neuronal activity in the hippocampus. In situ hybridization for c-fos was used to monitor the spread of neuronal activity in the brain; shown are representative coronal sections at approx. Bregma −1.3 to −2.0 of control (A) and OiS-mouse brains (B–D). After exposure of mice to 10% octanal, control animals showed a sparse and randomly distributed population of cells expressing c-fos (A). Similar neural activity was observed in OiS-mice exposed to mineral oil (B) or to 10% octanal if mice exhibited no seizure-like symptoms (C). Mice that experienced strong seizures in response to octanal (D) displayed a robust increase in c-fos expression in piriform cortex (Pir) and other M/T cell target areas like the olfactory amygdala (Am). In addition, we always observed increased neuronal activity in many areas of the brain including pronounced activity in the hippocampus (Hip) including the dentate gyrus (D). Scale bar, 1 mm.

Since seizures result from an over-excitation of neurons and the spread of this excitation in the brain, we anticipated that mice exhibiting odor-induced seizures would show increased neuronal activity in the piriform cortex, the next major center in the olfactory pathway. The piriform cortex of control animals revealed scattered *c-fos* expression in a distributed subset of neurons after octanal exposure (Fig. 4A), consistent with previous results [13]–[15], [26]–[28]. As expected, OiS-mice that showed no signs of seizure when exposed to octanal exhibited a similar pattern of scattered *c-fos* expression in piriform cortex (Fig. 4B). In marked contrast, OiS mice that experienced strong seizures displayed robust *c-fos* expression in the piriform cortex and other regions of the forebrain (Fig. 4C, D) as well as the hippocampus (Fig. 5), reminiscent of previous reports on kindled seizures [29]–[32].

In OiS-mice, octanal exposure always induced strong OSN activation (*c-fos* expression, Fig. 2) but neural activity in the olfactory cortex and forebrain was tightly correlated with seizure symptoms (Figs. 4 & 5). Why do different trials produce such different outcomes? We reasoned that the sense of smell needs mechanisms to separate salient and rapidly varying input from background and perhaps selectively inhibits widespread but slowly changing olfactory activity. To test this idea, we used an olfactometer to apply a controlled flow of octanal to OiS-mice. Remarkably, seizures were never observed (0/7 mice) when a 0–5% octanal gradient was delivered over a period of 4 minutes. In contrast, rapid exposure to 5% octanal induced symptoms in all animals within the first 20 s (7/7 mice; 2 strong, 4 moderate, 1 mild).

**Figure 6:**
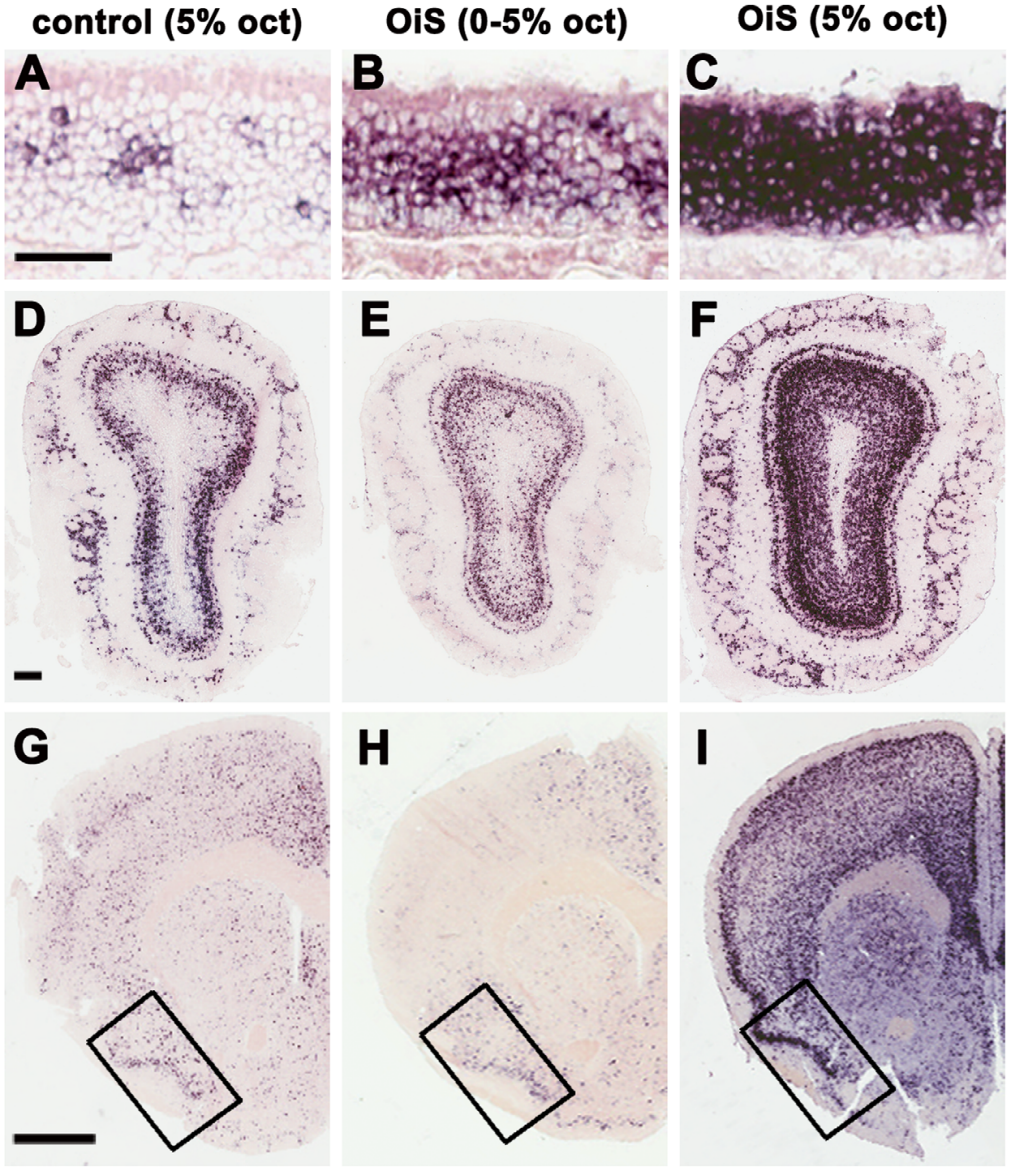
*c-fos* expression in OiS mice under controlled octanal exposure. Controlled delivery of octanal using an olfactometer results in neural activity patterns that closely resemble those observed when mice are passively exposed to odor. *In situ* hybridization for *c-fos* was used to monitor the spread of neuronal activity after exposure of mice to defined concentrations and gradients of octanal. Control mice exposed to 5% octanal for 1 minutes in the olfactometer (A, D, G) exhibit *c-fos* expression patterns in the MOE (A), MOB (D) and brain (G), including piriform cortex (boxed, G) that closely resemble those observed when control animals were passively exposed to odorant (Figs. 2–4). OiS mice exposed to a gradient of 0–5% octanal over a period of 4 minutes in the olfactometer show activation of the MOE (B) but little change in *c-fos* expression in the MOB (E) or brain (H). In contrast, OiS mice that were exposed to 5% octanal for 1 minute and exhibited strong seizures not only showed pronounced activation of the MOE (C) but also of M/T and GC cells in the MOB (F) as well as much of the forebrain including piriform cortex (I); see Fig. S3 for quantitation. Scale bars: A–C, 50 µm; D–F, 200 µm; G–I, 1 mm.

We also examined *c-fos* expression in mice from the two groups of mice that were exposed to octanal in the olfactometer. Fig. 6 demonstrates that in the olfactometer, both rapid and gradual exposure of OiS mice to octanal induces *c-fos* expression in almost every OSN. Interestingly, the level of *c-fos* expression induced by the octanal gradient was much lower than that observed after rapid delivery of 5% octanal indicating that peripheral desensitization occurs during this regime of gradually increasing odorant delivery. Importantly, however, both treatments activated a similar number of OSNs. Graded octanal delivery did not increase *c-fos* expression in the piriform (Fig. 6). In contrast, rapid exposure of OiS mice to 5% octanal activated M/T and granule cells in the MOB as well as piriform neurons and other regions of the forebrain (Figs. 6, S3). Thus controlled octanal delivery closely recapitulates the activity patterns observed when mice exhibited seizures after passive exposure to octanal and, as predicted, a rapid increase in global OSN activation can overcome powerful inhibitory processes that otherwise suppress widespread olfactory activity.

## Discussion

The OiS-line was unusual in that >90% of mature OSNs expressed *rI7* when crossed into the *OMP-TTA* background. More remarkable was the fact that octanal exposure triggered seizures in these animals with all mice exhibiting an epilepsy-like phenotype when exposed to a high concentration of odorant that was delivered rapidly. The variable symptoms (from mild to severe seizures) likely reflect competition between excitatory responses to odorant and the slightly slower, downstream recruitment of powerful feedback inhibition in the piriform cortex [13], [16], [17], [33]–[36] that control output to other brain regions. In marked contrast to effects of rapid octanal delivery, exposing OiS-mice to a gradually increasing concentration of this odorant never induced seizure despite activation of a similar number of OSNs (Fig. 6). Therefore it appears that the olfactory bulb acts as a gatekeeper by preventing signal from reaching the cortex whenever odorant is delivered slowly.

How general is this balance between excitatory and inhibitory responses in the MOB? The other line of mice expressing a functionally defined OR-transgene in all OSNs also exhibited strong attenuation of cognate odorant signaling at the level of the MOB [18]. Thus the OiS line is not unique in exhibiting strong inhibition in the olfactory bulb in response to widespread activation of OSNs. What role might this inhibition play in wild type mice? It is well known that as the concentration of an odorant increases, it activates an increasing number of distinct ORs and hence glomeruli; nonetheless many odorants retain their identity over wide concentration ranges. We suspect that strong inhibition of slowly changing responses in the MOB helps limit output to the most salient components. In the cortex, the recurrent excitatory network may have a role in pattern completion in response to weak signals from the MOB while the strong inhibitory network would prevent excessive spread of signal and help retain a consistent pattern of activity over a wide odor concentration range [16], [17]. We now show that when OiS mice are subjected to sudden and intense peripheral stimulation, inhibition of olfactory signaling [16]–[18] can be overcome or circumvented resulting in rapid spread of uncontrolled excitation and triggering a state of epilepsy-like seizure.

Epilepsy is a common broad spectrum disorder that remains uncontrolled in as many as 30% of patients receiving the best available medication [37]. Most widely used animal models of epilepsy require chemical injection or repeated electrical stimulation of the brain to trigger attacks. OiS-mice provide an interesting alternative model where seizures or seizure-related symptoms can be rapidly and reliably evoked simply by exposure to odorant. Importantly, the variable symptoms we observed in these animals after odorant exposure are highly reminiscent of the most common forms of human epilepsy where trigger events frequently produce a wide range of outcomes. Moreover, since the seizures in OiS-mice are directly dependent on olfactory input and thus initiated by a defined source of excitation, these animals present a uniform platform for studying mechanisms of epilepsy and may be useful for developing new therapeutic strategies.

## Materials and Methods

### Ethics Statement

All experimental procedures performed on mice described in this study were approved by the NIH/NIDCR Institutional Animal Care and Use Committee (IACUC) under protocols 8–495, 10–567 and 11–627.

### Transgenic Mice

All transgenic lines were produced by pronuclear injection of *tg(bi)OR* constructs into zygotes from FVB/N mice. Bidirectional TetO operator was generated by fusing another *CMV* minimal promoter to a standard *TetO* expression construct [38]. Thus, our bidirectional *TetO* operator consists of seven *TRE* flanked by two *CMV* minimal promoters. *tg(bi)OR* constructs utilized full-length *rI7* and *M72* sequences followed by either *IRES-GFP-SV40* poly(A) or *IRES-lacZ-BGH* poly(A). *OMP-TTA* mice have been described previously [20]. For all experiments, the transgenes were hemizygous and *OMP-TTA* knockin background heterozygous. Mice were of mixed FVB/N, C57BL, 129/Sv background. Littermates, without the *OMP-TTA* driver and *tg(bi)OR* transgene, or carrying only one of these transgenes, were used as controls.

Fifteen lines of *tg(bi)OR* mice were generated and crossed into an *OMP-TTA* background; 14 of the 15 lines expressed transgenic ORs in a subset of OMP-TTA expressing cells (20–60% of OSNs), in line with previous data reported for standard *TetO-OR* transgenes [18], [19], [21]. None of these lines exhibited seizure type phenotypes when exposed to octanal or acetophenone. The fifteenth line (the OiS-line) expressed transgenic odorant receptors in the vast majority of OSNs and thus resembles the “monoclonal nose” [18] and MOR28 [21] lines that have been described earlier. The reason why a few *TetO-*driven *tgORs* are present in >90% of *OMP-TTA* expressing neurons while the majority of *TetO-tgOR* lines have a much more restricted pattern of transgene expression is unclear but may reflect the sites of transgene incorporation in the genome. It is also possible that transgene positional effects are important for the induction of seizures that we observe, given the unique phenotype exhibited by OiS mice. However, we consider this unlikely since tested mice were hemizygous for the transgene and many of the control animals were also hemizygous for this particular transgene; moreover all seizures were octanal dependent, demonstrating the need for activation of transgene expressing OSNs.

### Odorant Exposure

For passive odorant exposure experiments, mice (at least 7 weeks old) were exposed to 20 µl of 1% or 10% odorant spotted on a filter paper in a 32×16×14 cm standard mouse cage for 3 minutes. All experiments were video recorded and seizure symptoms were categorized into three classes: weak, moderate, and strong. Mice that exhibited rapid blinking of eyes and temporarily lose function of their forelimbs resulting in their collapsing were classified as showing weak symptoms. For the moderate category, mice showed partial clonic seizures involving the head and forelimbs in addition to the symptoms associated with weak seizures. Mice scored as showing moderate seizures often also foamed at the mouth. The strong seizure category consisted of mice exhibiting maximal tonic-clonic seizures. A 4-channel olfactometer (KNOSYS) was used to deliver a defined odorant concentration to a small chamber (12 cm in diameter, 10 cm long). Animals were either exposed to air from the headspace above a gradually increasing concentration of octanal dissolved in mineral oil (0.05%, 0.5%, 2.5%, and 5% for 1 min each) or to a single concentration (5% for 1 min). Odorants were of the highest purity available from Sigma and were dissolved in mineral oil.

Habituation-dishabituation assays to investigate odor novelty were performed as described [22]. Briefly, male mice at least 7 weeks old were allowed to habituate to a standard mouse cage, and then a filter paper spotted with 20 µl of mineral oil was presented for 3 minutes. This was repeated three more times with 1 minute interval. On the fourth trial, 20 µl of the test odorant was presented for 3 minutes. All experiments were video recorded and investigation times for the third and fourth trials were measured. An investigation is defined as the nasal contact with the filter paper. At least 8 animals of each genotype were tested. Mice were used only once to avoid influencing data through learning.

### Histology and Fluorescence Imaging

*In situ* hybridization was performed as described previously [39]; ORs and *c-fos* were detected using full-length cRNA probes. For *c-fos* induction, mice were placed in a mouse cage and a filter paper spotted with 20 µl of either mineral oil or 10% octanal in mineral oil was introduced; after 30 minutes, tissue was harvested and frozen for *in situ* analysis. Multiple sections through the MOE, MOB, and brain were examined from at least three animals for each seizure category and control condition. We also noted that exposure of mice to 5 minutes of octanal in the olfactometer produced indistinguishable patterns of *c-fos* induction. Double-label *in situ* hybridization was carried out on 10 µm frozen sections using digoxigenin- and fluorescein-labeled antisense RNA probes as described [40]. Brightfield photomicrographs were obtained using either a Nikon Axiophot or a Scanscope CS system (Aperio). Confocal microscopy (1 µm optical sections) was performed with a Leica TCS SP2 (Leica Microsystems).

### Cell Count

Multiple sections (at least 4) from each animal from each condition were used to quantify *c-fos* positive cells from the anterior piriform cortex and PG and M/T cells in the MOB. All stained PG and M/T cells from each section were counted using the Aperio image analysis software nuclear stain algorithm. ImageJ was used to analyze stained cells in the anterior piriform cortex because the high density of cells in the strong seizure animals could not be resolved using the Aperio software.

### Statistical Analysis

Statistical analysis was performed in GraphPad Prism (GraphPad Software). *p* values for passive exposure experiments were calculated using one-way ANOVA with Newman-Keuls posterior test to compare *c-fos* positive cells from different conditions. For active odorant exposure experiments, *p* values were calculated using two-tailed, unpaired Student’s t-tests. *p* values for habituation-dishabituation assays were calculated using paired, two-tailed Student’s *t*-tests to compare differences in investigation time between mineral oil and specific odorant.

## Supporting Information

Figure S1 **Feedback control of OR-expression extends to receptors driven from the same promoter.** The distinct expression patterns of *rI7* and *M72* in OiS mice suggests that receptor feedback mechanisms extend to controlling expression of two transgenic ORs driven from the same synthetic promoter. To further investigate this, we performed two-color double-label *in situ* hybridization. Indeed cells expressing *M72* (A) rarely contained detectable *rI7* (B) and vice-versa; (C) shows the superimposed double labeled image demonstrating the presence of purple (*M72*) positive cells and green (*rI7*) positive cells but the virtual absence of double labeled OSNs; scale bar: 50 µm.
(TIF)

Figure S2 **OiS-mice show limited behavioral response to octanal.** A standard habituation-dishabituation assay was used to assess odor novelty and significance of a range of stimuli including the acetophenone, a cognate odorant for the M72 receptor and octanal, which activates the rI7 receptor. Both control and OiS mice showed significantly increased investigation times when presented with female mouse urine as odorant relative to the time that they investigate the carrier mineral oil (dotted black line); however, control mice investigated this stimulus for about 3-times as long as OiS mice. Interestingly OiS (but not control) mice reacted to acetophenone as though it might represent a significantly novel stimulus but neither group showed extended investigation of octanal or other odorants in this assay. Since it is unlikely that control mice are unable to distinguish these odorants from mineral oil, these data demonstrate the limited utility of this assay. It should be noted that this assay is performed on naïve animals where most odors are not thought to have attractive or aversive valance. However, the highly extended investigation of mouse urine is likely related to relevance of this cue to mice and possibly activation of hardwired circuits. All odorants, except for mouse and fox urine, presented undiluted) were used at 1% dissolved in mineral oil. *denotes *p*<0.05, **,*p*<0.01, ***,*p*<0.001.
(TIF)

Figure S3 **Quantitation of** ***c-fos*** **induction in response to controlled delivery of 5% octanal in the MOB and piriform cortex.** *In situ* hybridization for *c-fos* was used to monitor the spread of neuronal activity after exposure of mice to 5% octanal for 1 minute in an olfactometer. Under these conditions OiS mice reliably showed symptoms of seizure whereas control mice did not exhibit unusual behavioral responses. Fig. 6 illustrates the differences in *c-fos* induction observed between these genotypes. Quantitation of the number of *c-fos* positive cells was performed to investigate differences in activation of the inhibitory PG and excitatory M/T cells in the MOB as well as piriform cortex neurons in control and OiS mice that exhibited strong seizures. Notably, OiS mice exhibit significantly more activity in M/T and piriform neurons than control animals under these conditions. Thus in OiS mice, strong seizures induced by rapid octanal delivery are associated with neural activity that closely resembles the pattern observed in mice that exhibited similar symptoms when presented with a much less well defined olfactory stimulus in a modified habituation-dishabituation assay (Figs. 3, 4); data are mean ± s.e.m., n = 3 animals for control; n = 2 for OiS; *denotes *p*<0.05.
(TIF)

Movie S1 **OiS-mouse exhibiting strong seizure-like symptoms in the presence of octanal.**
(WMV)

Movie S2 **OiS-mouse exhibiting moderate seizure-like symptoms in the presence of octanal.**
(WMV)

Movie S3 **OiS-mouse exhibiting weak seizure-like symptoms in the presence of octanal.**
(WMV)

Movie S4 **Control-mouse exposed to octanal (no seizure-like symptoms).**
(WMV)

Movie S5 **OiS-mouse exposed to acetophenone (no seizure-like symptoms).**
(WMV)

Movie S6 **OiS-mouse exhibiting no seizure-like symptoms in the presence of octanal.**
(WMV)
