# Supplementary figures and images for: A Smell That Causes Seizure
Source: PLoS One. 2012 Jul 27;7(7):e41899. doi: 10.1371/journal.pone.0041899 (PMC3407102; doi:10.1371/journal.pone.0041899)

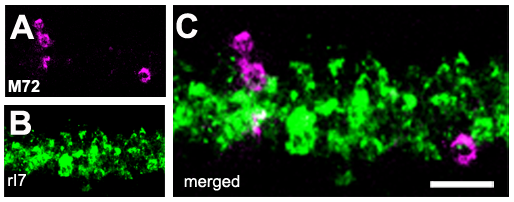

Supplement: Figure S1 — Feedback control of OR-expression extends to receptors driven from the same promoter. The distinct expression patterns of rI7 and M72 in OiS mice suggests that receptor feedback mechanisms extend to controlling expression of two transgenic ORs driven from the same synthetic promoter. To further investigate this, we performed two-color double-label in situ hybridization. Indeed cells expressing M72 (A) rarely contained detectable rI7 (B) and vice-versa; (C) shows the superimposed double labeled image demonstrating the presence of purple (M72) positive cells and green (rI7) positive cells but the virtual absence of double labeled OSNs; scale bar: 50 µm. (TIF) [file pone.0041899.s001.tif]

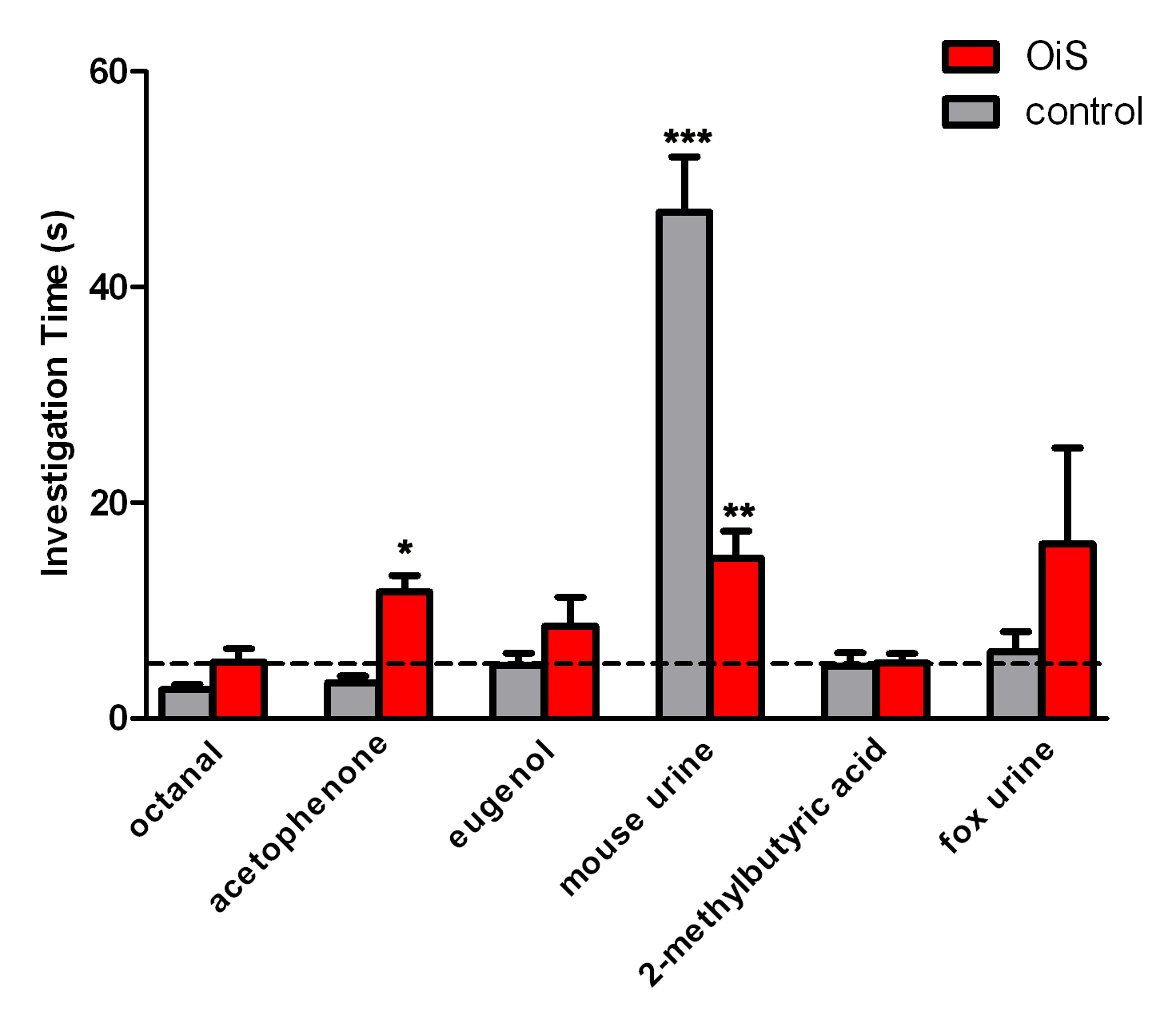

Supplement: Figure S2 — OiS-mice show limited behavioral response to octanal. A standard habituation-dishabituation assay was used to assess odor novelty and significance of a range of stimuli including the acetophenone, a cognate odorant for the M72 receptor and octanal, which activates the rI7 receptor. Both control and OiS mice showed significantly increased investigation times when presented with female mouse urine as odorant relative to the time that they investigate the carrier mineral oil (dotted black line); however, control mice investigated this stimulus for about 3-times as long as OiS mice. Interestingly OiS (but not control) mice reacted to acetophenone as though it might represent a significantly novel stimulus but neither group showed extended investigation of octanal or other odorants in this assay. Since it is unlikely that control mice are unable to distinguish these odorants from mineral oil, these data demonstrate the limited utility of this assay. It should be noted that this assay is performed on naïve animals where most odors are not thought to have attractive or aversive valance. However, the highly extended investigation of mouse urine is likely related to relevance of this cue to mice and possibly activation of hardwired circuits. All odorants, except for mouse and fox urine, presented undiluted) were used at 1% dissolved in mineral oil. *denotes p<0.05, **,p<0.01, ***,p<0.001. (TIF) [file pone.0041899.s002.tif]

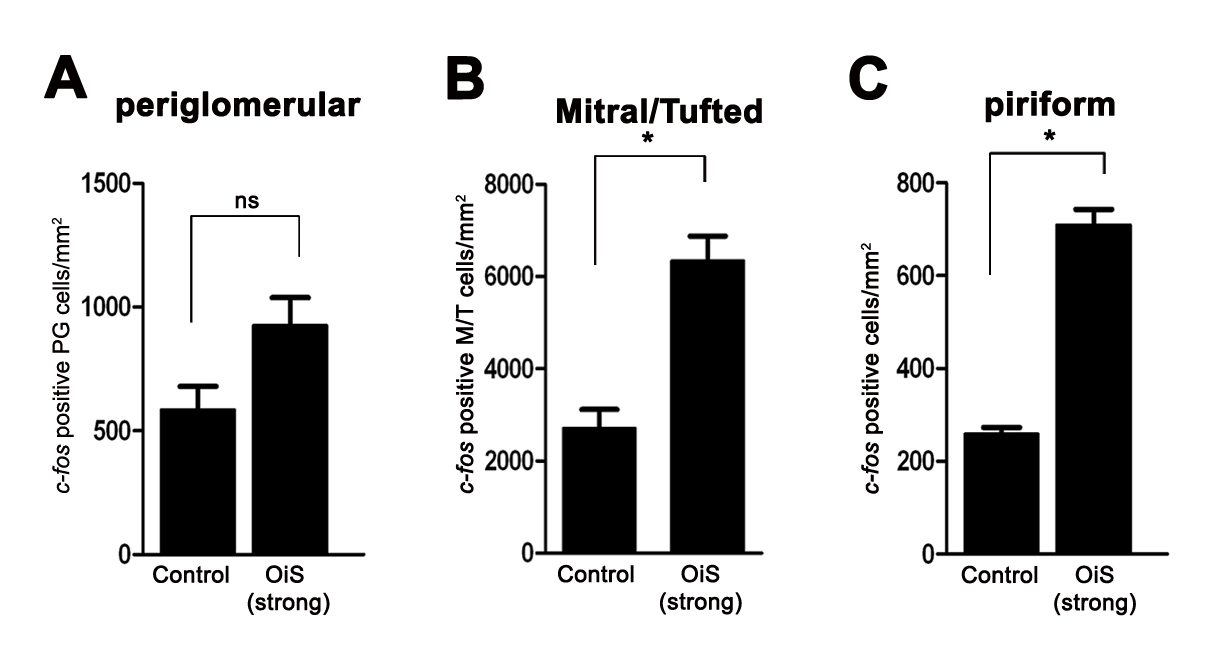

Supplement: Figure S3 — Quantitation of c-fos induction in response to controlled delivery of 5% octanal in the MOB and piriform cortex. In situ hybridization for c-fos was used to monitor the spread of neuronal activity after exposure of mice to 5% octanal for 1 minute in an olfactometer. Under these conditions OiS mice reliably showed symptoms of seizure whereas control mice did not exhibit unusual behavioral responses. Fig. 6 illustrates the differences in c-fos induction observed between these genotypes. Quantitation of the number of c-fos positive cells was performed to investigate differences in activation of the inhibitory PG and excitatory M/T cells in the MOB as well as piriform cortex neurons in control and OiS mice that exhibited strong seizures. Notably, OiS mice exhibit significantly more activity in M/T and piriform neurons than control animals under these conditions. Thus in OiS mice, strong seizures induced by rapid octanal delivery are associated with neural activity that closely resembles the pattern observed in mice that exhibited similar symptoms when presented with a much less well defined olfactory stimulus in a modified habituation-dishabituation assay (Figs. 3, 4); data are mean ± s.e.m., n = 3 animals for control; n = 2 for OiS; *denotes p<0.05. (TIF) [file pone.0041899.s003.tif]
